# Pre- and Post-Neoadjuvant Clinicopathological Parameters Can Help in the Prognosis and the Prediction of Response in HER2+ and Triple Negative Breast Cancer

**DOI:** 10.3390/cancers15123068

**Published:** 2023-06-06

**Authors:** Laura Pons, Laura Hernández, Aintzane Urbizu, Paula Osorio, Paula Rodríguez-Martínez, Eva Castella, Ana Muñoz, Carolina Sanz, Laura Arnaldo, Eudald Felip, Vanesa Quiroga, Gustavo Tapia, Mireia Margelí, Pedro Luis Fernandez

**Affiliations:** 1 Department of Pathology, Germans Trias i Pujol Universitary Hospital, Institut Germans Trias i Pujol (IGTP), 08916 Badalona, Spain; lponsmar.germanstrias@gencat.cat (L.P.); lhernandezl.germanstrias@gencat.cat (L.H.); aurbizus.germanstrias@gencat.cat (A.U.); posorioer.germanstrias@gencat.cat (P.O.); prodriguez.germanstrias@gencat.cat (P.R.-M.); ecastella.germanstrias@gencat.cat (E.C.); ammunoz.germanstrias@gencat.cat (A.M.); carosanz.germanstrias@gencat.cat (C.S.); larnaldoo.germanstrias@gencat.cat (L.A.); gtapia.germanstrias@gencat.cat (G.T.); 2 Medical Oncology Department, Catalan Institute of Oncology, B-ARGO Groups, Institut Germans Trias i Pujol (IGTP), 18916 Badalona, Spain; efelip@iconcologia.net (E.F.); vquiroga@iconcologia.net (V.Q.); mmargeli@iconcologia.net (M.M.); 3 Faculty of Medicine and Health Sciences, Universitat Autonoma de Barcelona, 08193 Barcelona, Spain

**Keywords:** breast cancer, neoadjuvant chemotherapy, HER2+, triple negative

## Abstract

**Simple Summary:**

HER2+ and triple negative breast cancers are widely known for their aggressiveness, frequent resistance to treatment and poor prognosis. Neoadjuvant management has provided promising results for both subtypes, but there is still a subset of patients with no or low response. Consequently, a non-negligible number of patients is receiving a treatment regimen that might not be adequate. Identification of these patients is essential to avoid overtreatment and provide more effective treatment options.

**Abstract:**

Neoadjuvant treatment (NAT) is one of the most widely used options for HER2+ and triple negative (TN) early breast cancer (BC). Since around half of the patients treated with NAT do not achieve a pathologically complete response (pCR), biomarkers to predict resistance are urgently needed. The correlation of clinicopathological factors with pCR was studied in 150 patients (HER2 = 81; TN = 69) and pre- and post-NAT differences in tumour biomarkers were compared. Low estrogen receptor (ER) expression, high tumour-infiltrating lymphocytes (TILs) and low cT-stage were associated with pCR in HER2+ tumours (*p* = 0.022; *p* = 0.032 and *p* = 0.005, respectively). Furthermore, ER expression was also associated with residual cancer burden (RCB; *p* = 0.046) in the HER2+ subtype. Similarly, pre-NAT, low progesterone receptor expression (PR; 1–10%) was associated with higher RCB (*p* < 0.001) in TN tumours. Only clinical and pathological T-stage (cpT-stage) had prognostic capacity in HER2+ tumours, whereas pre-NAT cpT-stage and post-NAT TILs had this capacity for the prognosis of TN tumours. We conclude that ER and PR expression may help predict response to NAT in HER2 and TN BC and should be taken into account in residual tumours. Also, changes observed in the phenotype after NAT suggest the need to reevaluate biomarkers in surviving residual tumour cells.

## 1. Introduction

Neoadjuvant treatment (NAT) has become a useful therapeutic option to reduce tumoural size in locally advanced and frequently inoperable invasive breast cancer (IBC). Subsequently, its use has been extended to non-locally advanced tumours in order to increase breast preservation rates and to achieve pathological complete response (pCR) [1,2,3,4]. pCR to NAT is a prognostic factor itself that is based on histopathological evaluation of the tumour bed and lymph nodes in excision specimens. Achievement of pCR has demonstrated to be related to better outcomes in IBC, especially in HER2-positive (HER2+) and triple negative (TN) surrogate subtypes [5,6], which are most commonly treated with NAT. The rate of pCR slightly varies among published studies, reaching rates up to 40–75% in HER2+ tumours treated with dual HER2 therapy and 40–48% in TN tumours [7,8,9,10]. Considering that around half of the patients treated with NAT do not achieve pCR, there is an urgent need to find useful biomarkers to predict this resistance in order to identify likely non-responder patients in the pretreatment biopsy, and to determine the best therapeutic approach. Previous studies have shown that the Ki67 labelling index (LI), hormone receptor (HR) expression, stromal tumour infiltrating lymphocytes (TILs) or T-stage could be useful for this purpose [11,12,13,14]. We postulated that, besides pre-NAT biomarkers, characterisation of residual tumours in non-responder patients and comparison with their pre-treatment counterparts could contribute to better knowledge of the mechanisms involved in NAT resistance and help in the prediction and prognosis of BC.

## 2. Materials and Methods

### 2.1. Study Design

A total of 150 patients diagnosed with HER2+ and TN IBC treated with NAT from 2016 to 2022 were collected based on the following criteria: (1) age above 18 years, (2) treated with at least four cycles of systemic treatment, (3) availability of pre-treatment biopsy and post-treatment specimen, and (4) availability of informed consent. We identified 81 HER2+ and 69 TN IBC. Clinicopathological data such as age at diagnosis, histological grade, HR status, Ki67 expression, percentage of TILs, treatment regimen or stage were obtained for each patient. All cases were reviewed by a pathologist specialized in çbreast disease. Samples with an extremely low representation of tumour cells (less than 10 high-power fields) or the impossibility of review by our specialized pathologist were excluded from of the particular analyses and classified as “Not available”. Staging was performed according to the American Joint Committee on Cancer guidelines [15].

Clinicopathological characteristics such as histological grade, HR expression, Ki67 LI, TILs, T-stage and N-stage were analysed in association with pCR in order to find a useful biomarker to predict response to NAT. These variables were also correlated with residual cancer burden (RCB), another method to measure residual disease for obtaining long-term prognostic information [16]. The online MD Anderson calculator provides an RCB index, which is derived from the largest area and cellularity of residual invasive primary cancer, the number of positive lymph nodes and size of the largest metastasis [17]. A residual tumour was subsequently classified as inexistent (RCB-0), minimal (RCB-I), moderate (RCB-II) or extensive (RCB-III).

To characterise the changes in tumour cells due to NAT, primary (pre-NAT) and residual (post-NAT) histopathological characteristics were compared in cases with no pCR. The surrogate subtypes derived from the immunohistochemical study of primary and residual tumours were also compared.

### 2.2. Pathology Assessment

Oestrogen receptor (ER) and progesterone receptor (PR) status was assessed by immunohistochemistry (IHC) and was considered negative if less than 1% of cells were stained positively by IHC. HER2 positivity was defined as either HER2 expression by IHC (score 3+) or gene amplification by fluorescent in situ hybridation (FISH) (in cases with a score 2+ by IHC) according to the American Society of Clinical Oncology/College of American Pathologists (ASCO/CAP) guidelines [18]. TN tumours were defined as those with ER and PR expression of less than 1% of positive cells plus lack of HER2 positivity. We also included tumours with low HR expression (1–10%) and HER2− in the TN group to evaluate a possible difference in behaviour, given that they are commonly treated as TN. HER2+ tumours were defined as HER2+, irrespective of hormonal receptor expression. Ki67 LI was evaluated following the guidelines of the International Ki67 Working Group [19]. The four biomarkers (ER, PR, HER2, Ki67) were evaluated in all pre-treated samples (core needle biopsy) and in all post-treated specimens (lumpectomy or mastectomy) with residual tumour. These biomarkers were used to classify tumours into the surrogate subtypes according to the St. Gallen International Expert Consensus of 2017 [20]. HER2+ and TN cancers are highly proliferative, and thus, Ki67 LI was classified as low (<30%) and high (≥30%) to distinguish truly proliferative from somewhat proliferative tumours [19,21]. TILs were assessed according to the guidelines of the International TILs Working Group [22,23] and classified as low (≤20%) and high (>20%). pCR was determined by exhaustive microscopic examination of the excised tumour bed and lymph nodes after completion of NAT. Evaluation of sentinel nodes was performed in all cases. We included ypT0 ypN0 and ypTis ypN0 as acceptable definitions of pCR.

### 2.3. Statistical Analyses

The statistical analysis was performed with SPSS (version 24; SPSS Inc., Chicago, IL, USA) and R software (version 4.0.3 for Windows). The clinicopathological characteristics were compared using a Fisher’s exact test for categorical variables (histological grade, ER, PR, HER2 status, high and low-TILs, Ki67 subgroups, T-stage and N-stage) and a Student’s *t* test for continuous variables (age, Ki67 and TILs means). To test for normality, the Shapiro–Wilk test was performed on the continuous data. Multivariate analysis was carried out using a logistic regression method to create a general linear model, in which the dependent variable was pCR. Relapse-free survival (RFS) was estimated using the Kaplan–Meier method and compared using the log-rank test.

## 3. Results

### 3.1. Clinicopathological Parameters

The mean age of the 150 IBC patients studied was 53.5 years (range 26–77) and the median follow-up was 26 months. Most of the tumours were of no special type (HER2+ = 97.5%; TN = 97.1%). Among the HER2+ tumours, histological grade 2 was the most common (50.6%), whereas histological grade 3 was the most frequent in TN tumours (79.2%). The mean Ki67 LI was higher in TN (62%) than in HER2+ tumours (37.9%). There were more tumours with a high percentage of TILs in the TN (34.5%) than in the HER2+ subtype (22.6%). Both subtypes were most frequently classified as T2 by clinical T-stage (HER2+ = 65.4%; TN = 71%), but differed in the clinical N-stage (N0 HER2+ = 42%; N0 TN = 62.3%).

The treatment regimen most frequently used in HER2+ patients was anthracyclines plus trastuzumab-pertuzumab-paclitaxel (THP; *n* = 62; 76.5%). Other treatment regimens were docetaxel-carboplatin-pertuzumab-trastuzumab, THP alone or THP plus atezolizumab (anti-PD-L1). The most frequent neoadjuvant regimen in TN tumours was paclitaxel followed by epirubicin-cyclophosphamamide (*n* = 34; 49.3%). Table 1 shows the distribution of all the treatment regimens and the correlation with pCR. No significant differences were found in response to the treatment regimen between the two subtypes.

### 3.2. Response to Neoadjuvant Treatment

Comparison of the histopathological characteristics between the responders (achievement of pCR) and non-responders (no achievement of pCR) is shown in Table 2. pCR was achieved in 39 HER2+ patients (48.1%) and 37 TN patients (53.6%).

**Figure 1:**
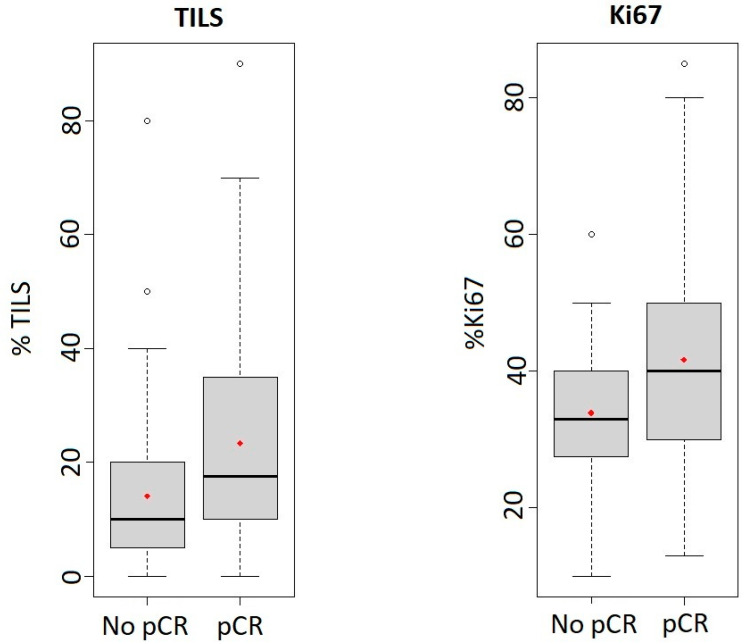
Box plots of TILs (pCR *n* = 38; No pCR *n* = 40) and Ki67 (pCR *n* = 38; No pCR *n* = 35) expression in HER2+ tumours in responder and non-responder patients. Expression is represented as a percentage, the median is shown as the black line within the interquartile box and the central dot is the mean.

Among HER2+ patients, responder tumours were more frequently classified as high grade (48.7%) while non-responder tumours were mainly grade 1/2 (61.9%). A significantly greater proportion of non-responders showed ER expression (76.2%; *p* = 0.022). In contrast, a similar proportion of PR expression was found in both groups. The mean Ki67 LI was higher in responders than in non-responders, and this difference was statistically significant (*p* = 0.022). A high Ki67 LI (≥30%) was observed in 76.9% of responders and in 52.4% of non-responders, although this difference was not statistically significant. Lymphocytic infiltration showed a significant association with pCR in the HER2+ subtype, since 33.3% of responders had a high percentage of TILs and only 11.9% of non-responders was classified in this group (*p* = 0.032). Indeed, multivariate analysis demonstrated that the percentage of TILs was an independent prognostic factor for pCR in the HER2+ subtype. Figure 1 shows obvious differences between box plots for TILs and Ki67 in responder and non-responder patients. Not unexpectedly, tumour size, expressed as T-stage, was found to be significantly correlated with pCR in HER2+ tumours, since only 10.3% of T3/T4 patients were responders.

**Figure 2:**
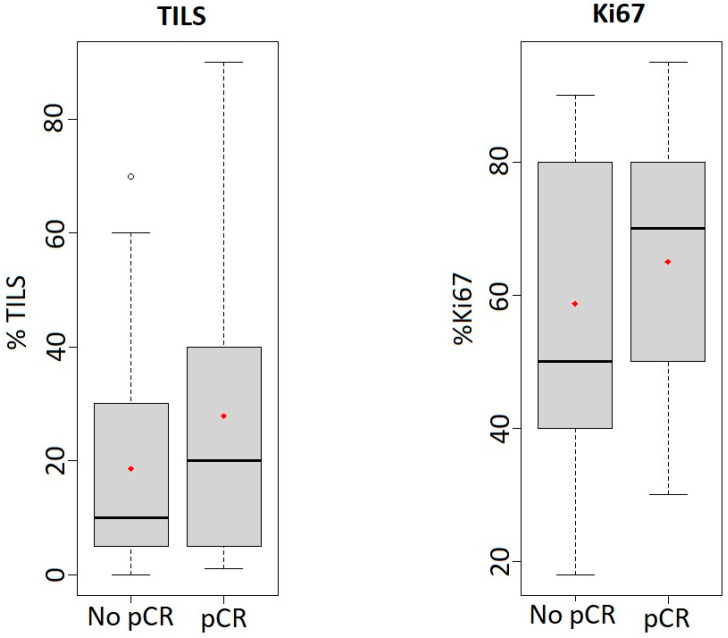
Box plots of TILs (pCR = 36; No pCR = 31) and Ki67 (pCR = 35; No pCR = 29) expression in TN tumours in responder and non-responder patients. Expression is represented in percentage; the median is shown as the black line within the interquartile box and the central dot is the mean.

Among TN tumours, both responder and non-responder tumours were ER negative (94.6% and 96.9%, respectively) and PR negative (86.5% and 81.3%). Responders presented mostly as high grade tumours (86.5%), and were not significantly different in this respect from non-responders (*p* = 0.097). Ki67 LI was higher in responders than in non-responders, but this association was not statistically significant. Surprisingly, although there was a difference in the mean number of TILs in responders and non-responders, these differences were not statistically significant (*p* = 0.111). Figure 2 also shows the differences in TILs and Ki67 expression between the box plots of responders and non-responders. Contrary to HER2+ tumours, there was no statistical correlation between T-stage and pCR.

Table 3 shows the correlation of clinicohistopathological variables with RCB in the HER2+ subtype. Consistent with the results shown in Table 1, we found that 80.8% of HER2+ patients with high residual tumour (RCB-II/III) were ER positive, which contributed to the association between ER expression and RCB (*p* = 0.046).

Table 4 shows the same correlation of variables in TN tumours. Interestingly, we found a significant association between PR expression and RCB, in part, because most of the tumours with RCB-II/III showed PR expression (57.9%; *p* < 0.001).

**Figure 3:**
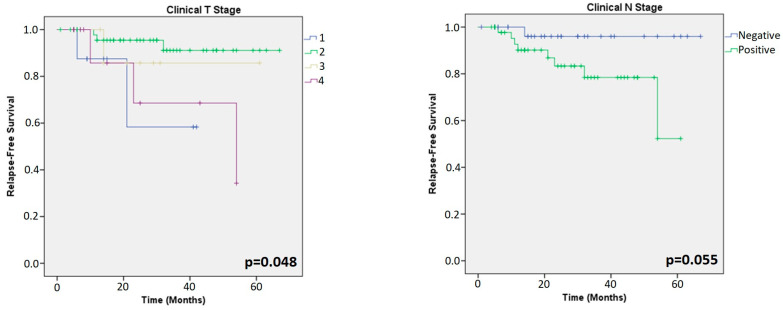
Kaplan–Meier analysis for prognosis based on clinical T- and N-stage of the primary tumours in HER2+ patients.

**Figure 4:**
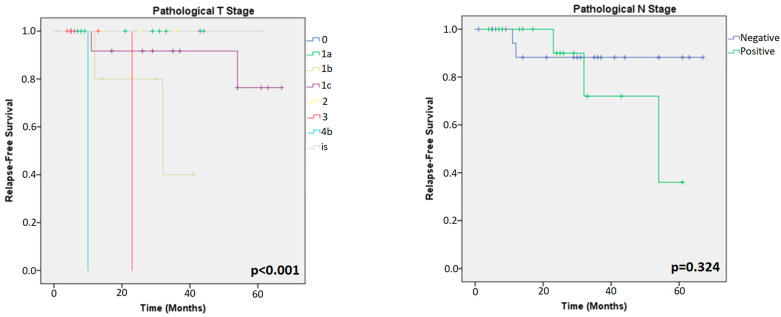
Kaplan–Meier analysis for prognosis based on parameters of residual tumours in HER2+ patients.

All the patients (81 HER2+ and 69 TN) were included in the Kaplan–Meier survival analysis. Among HER2+ tumours, the analysis showed that clinical and pathological T stages (cpT-stage) were the only significant factors related to RFS (*p* = 0.048; *p* < 0.001, respectively) (Figure 3 and Figure 4). ER, PR, Ki67 (in primary and residual tumour), TILs (in primary and residual tumours) and clinical/pathological N stage showed similar survival curves across all groups in HER2+ patients.

**Figure 5:**
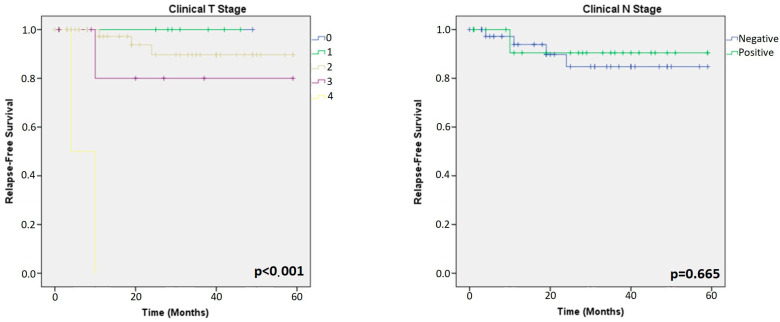
Kaplan–Meier analysis for prognosis of primary tumours in TN patients.

**Figure 6:**
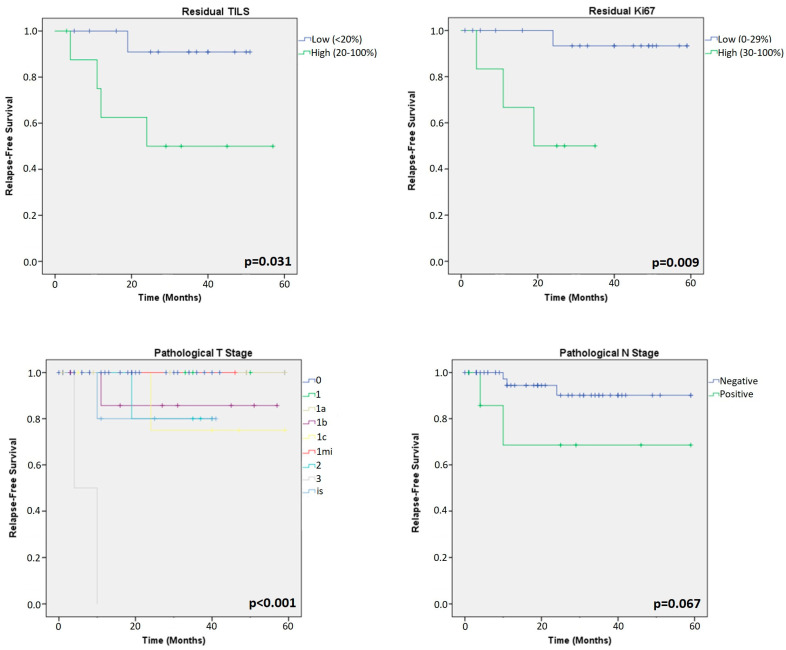
Kaplan–Meier analysis for prognosis of residual tumours in TN patients.

Among TN tumours, the analysis showed that cpT-stages were also a prognostic factor for RFS (*p* < 0.001; *p* < 0.001, respectively). Interestingly, the analysis of residual tumours showed that low TILs and low Ki67 LI were prognostic factors for RFS (*p* = 0.031 and *p* = 0.009, respectively), as shown in Figure 5 and Figure 6.

### 3.3. Comparision of Primary and Residual Tumors in Non-Responder Patients

To characterise the histopathological changes associated with response to NAT, we compared the clinicopathological characteristics of primary and residual tumors in non-responder patients (Table 5).

**Figure 7:**
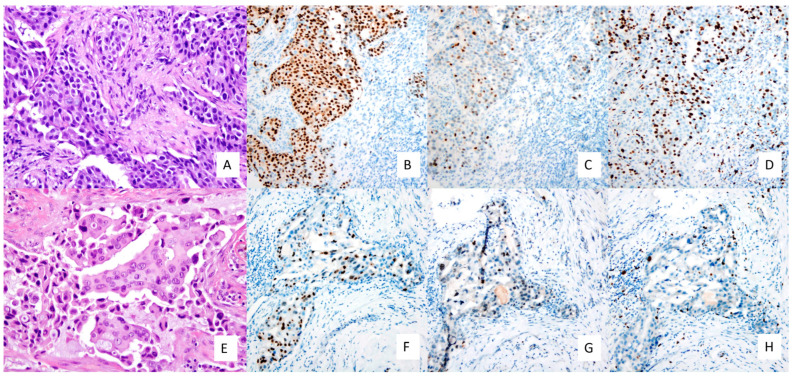
Histological and IHC differences between primary and residual HER2+ tumours. (**A**) Primary tumour showing histological grade 3 and low TILs (×200), (**B**) ER expression of 90% (×100), (**C**) PR expression of 20% (×100), (**D**) Ki67 of 45% (×100); and residual tumour showing (**E**) histological grade 2 and low TILs (×200), (**F**) ER expression of 50% (×100), (**G**) PR expression of 10% (×100) and (**H**) Ki67 of 10% (×100).

A decrease, albeit not significant, in histological grade was observed in residual tumour cells of HER2+ tumours. Lack of significance could be related to the number of non-gradable residual tumours (isolated tumour cells). The percentage of ER and PR expression was significantly decreased in residual HER2+ tumours (*p* = 0.021 and *p* = 0.021, respectively). HER2 status was also significantly modified by NAT, showing a loss of HER2 positivity in 19.1% of cases (*p* = 0.001). The Ki67 LI and mean also showed a significant reduction in residual tumours (*p* = 0.007). The decrease in histological grade, ER and PR expression and Ki67 percentage is shown in Figure 7. There were no significant differences between the percentage of TILs in responder and non-responder patients. As expected, T-stage (clinical for primary and pathological for residual) significantly decreased in residual tumours, with most of the primary tumours classified as T2, whereas most of the residual tumours were classified as T0/T1 (*p* < 0.001). Although not significant, we observed a change in nodal status, in which the percentage of positive lymph nodes reduced from primary to residual tumours (61.9% vs. 40.5%).

Among TN tumours, a decrease in histological grade was observed, but without significance. The mean Ki67 also showed a significant reduction in residual tumours (56% vs. 17.8%; *p* < 0.001). Similarly, T-stage decreased in residual tumours with most of the primary tumours classified as T2, whereas most of the residual tumours were classified as T0/T1 (*p* < 0.001). We found no significant correlation between pCR and the rest of clinicopathological parameters (HR expression, HER2 status, Ki67 LI, TILs or N-stage) in TN tumours.

**Figure 8:**
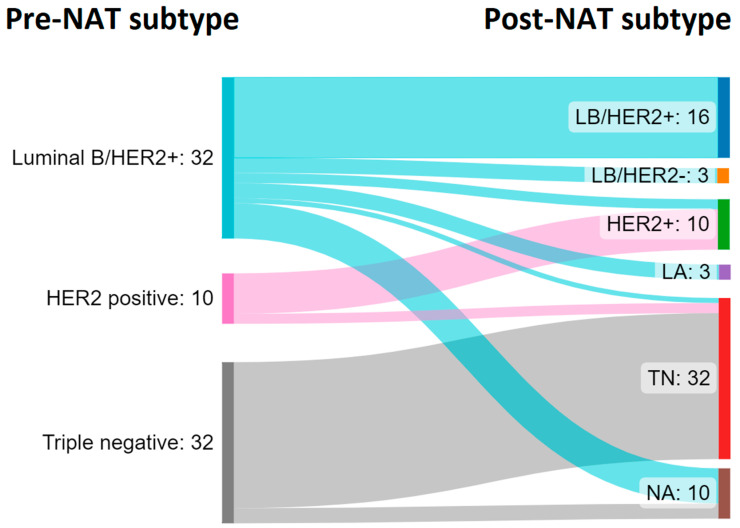
Sankey diagram showing flow distribution from pre-NAT to post-NAT subtypes derived from IHC analysis.

The IHC evaluation of residual tumours allowed comparison of the surrogate subtypes. Among the HER2+ non-responder patients (*n* = 42), there were 32 luminal B/HER2+ and 10 HER2+. In addition, among the pre-NAT luminal B-like HER2+ tumors (*n* = 32), 16 cases maintained the same intrinsic surrogate subtype; 3 cases became luminal B-like HER2−; 2 cases changed into HER2+; 1 case changed into TN and 3 cases changed into luminal A-like. There was not sufficient material to perform the assays of the four biomarkers or FISH in 7 cases. Among pre-NAT HER2+ tumours (*n* = 10), 8 cases mantained the same intrinsic surrogate subtype and 2 cases became TN in post-NAT residual tumour cells. The cases classified as TN in pre-NAT samples were all classified as TN in the post-NAT resection specimen, with the exception of 3 cases for which sufficient residual tumour cells were not available to perform IHC. The flow of cases is shown in Figure 8. No HER2+ cases becoming HR positive after NAT were found.

## 4. Discussion

In this study a thorough analysis of the possible influence of clinicopathological parameters in response to NAT and the phenotypical evolution of these tumours was performed.

### 4.1. Predictive and Prognostic Biomarkers in HER2+ and TN Tumours

#### 4.1.1. Histological Grade

There are contradictory data in the literature regarding the influence of the histological grade with respect to NAT response. The results of the Early Breast Cancer Trialists’ Collaborative Group (EBCTCG) suggest that the reduction in distant recurrences due to chemotherapy is the same regardless of histological grade [24]. On the other hand, Jarzab et al. analysed 353 BC patients treated with chemotherapy and showed that grade 3 tumours exhibited a higher proportion of pCR, concluding that histological grade was an independent predictor of pCR [25]. Other studies including a specific HER2 cohort also described this correlation [12,26]. Our work did not find this association to be significant, supporting the findings of the EBCTCG, despite a higher proportion of HER2+ histological grade 3 tumours in responders than in non-responders. Regarding TN tumours, another study showed the correlation of histological grade and pCR [27]. Again, although we could not statistically confirm this relationship, a higher proportion of grade 2 was observed in non-responders in our study (25% vs. 5.8%).

#### 4.1.2. Hormone Receptors

Another important histopathological factor related to pCR is HR status. The study conducted by Kurozumi et al. found a statistically significant correlation between HR and pCR in HER2+ patients [12]. Similar results were found by Petit et al. in a cohort of 177 patients, showing that only ER and Ki67 were predictive factors of pCR in HR-positive BC. Our work also shows that the negativity for ER is not only correlated with higher rates of pCR, but also with RCB-0/I in the HER2+ subtype, similar to the results reported by Meisel et al. [28]. In conjunction with previous publications, our results reinforce the idea that ER+/HER2+ tumours present a lower probability of achieving pCR.

Our study also shows that the expression of PR is correlated with RCB-II/III in TN tumours; in other words, patients with low expression of PR (1–10%) respond worse to NAT than those with no expression of PR. Our results suggest that this hormonal influence could derive into a more aggressive behaviour in this type of BC.

#### 4.1.3. Ki67 LI

Ki67 LI is an indicator of cellular proliferation, evaluated in multiple studies as a predictor of pCR in the neoadjuvant context, with most reporting a statistically significant association [12,13,27,28,29,30,31,32,33,34]. Our study also found differences but they did not reach statistical significance. This is in line with the most recent recommendations of the International Ki67 Working Group, which does not support the use of Ki67 as a predictor of efficacy of chemotherapy [19]. Ki67 as a prognostic factor has also been analysed in several studies [35,36,37,38]. The study of Li et al. included more than 14,000 patients and concluded that Ki67 quantification (both pre-NAT and post-NAT) could predict prognosis in breast cancer patients [39]. Our study shows that the Ki67 post-NAT could be a prognostic factor in TN patients. This is not surprising and could be explained as less proliferative subclones, selected by NAT, are less aggressive and consequently, patients have a better prognosis. These results highlight the importance of re-evaluating this marker in residual tumours.

#### 4.1.4. TILs

According to the previous studies of Denkert et al. published in 2010 and 2019 [11,40], pre-NAT TILs are predictive of response to chemotherapy in all IBC subtypes, especially in TN and HER2+ tumours. Our results coincide with these publications, showing a significant correlation between TILs and pCR in HER2+ tumours. More specifically, 25% of responders had high-TILs values compared to only 7.1% of non-responders. We found a similar trend in TN tumours (27% responders vs. 12.5% non-responders), although it did not achieve statistical significance in this subtype. The association between high infiltration TILs and pCR is widely known, but the prognostic value of TILs infiltration in residual disease post-NAT is controversial. In some reports, a decrease or increase in TILs corresponded to a better disease-free survival as compared, to unchanged levels that were associated with a worse prognosis [41]. Our analysis showed an improved prognosis for RFS associated with low TILs in residual TN tumours. These results support the idea that providing this information both in pre-NAT biopsy and post-NAT specimens could be useful in patient management.

#### 4.1.5. T-Stage

Tumoral size, expressed as T-stage, has previously been studied as a predictor of pCR in BC [42,43]. The study by Prat et al. [44] including 957 patients with BC showed that T-stage was the most significant variable associated with pCR. Another study of 2366 patients showed similar results [14]. Our study also shows this important and significant relationship in the HER2+ subtype, but not in TN BC. Nonetheless, this parameter proved to have prognostic significance for RFS in both phenotypes.

### 4.2. Differences in Biomarkers in Primary and Residual Tumors

We hypothesized that the effect of NAT in cancer cells could cause phenotypical changes in the surviving clones. Indeed, our results showed when comparing primary and residual tumours in non-responder patients, there is a reduction in the percentage of ER and PR expression in cases with HER2+ tumours. This is in line with previous studies showing a decrease or even negativity of HR post-NAT [45,46,47]. We also observed a negative switch of HER2 status in 16.6% of HER2-residual tumors and no cases of positive transformation. The effect of NAT on HER2+ tumours has been studied previously, showing a loss of HER2 amplification within a range of 12–43% [47,48,49] as the most frequent change. Acccording to Grassini et al., it remains unclear if this change is due to response/resistance to therapy or to an underlying HER2 heterogeneity [50]. This is not surprising given that the few cells that co-express HER2 and HR are affected by the anti-HER2 therapy and, consequently, there is a concomitant reduction in HR expression. Nevertheless, even with the targeted anti-HER2 therapy, some subclones still do not respond, and can be detected by IHQ in residual tumours.

Ki67 LI variation between pre- and post-NAT has shown to predict prognosis in HER2+ and TN BC [51,52]. Our study showed a significant reduction in Ki67 LI due to NAT, which is in line with previous studies [53,54], supporting the idea of a direct action of the treatment on the more proliferative subclones, leading to better response in these cells and better prognosis for these patients.

Regarding the lymphocytic infiltration of cancers, TIL values showed a significant decrease in HER2+ tumours post-NAT, suggesting a possible anti-immunogenic effect of HER2-targeted therapy. We also observed a slight, although not statistically significant, decrease in TILs in residual TN tumours, which is in line with the study by Ochi et al., showing that the percentage of TILs did not change after NAT in 70.9% of TN BC [55]. This could be related to the known, highly immunogenic biology of TN tumours and the recent incorporation of immunotherapy in the NAT of TN tumours.

Post-NAT changes in biomarkers, such as HR, Ki67 and HER2, can have potential prognostic significance. For instance, a previous study proved that loss of HR expression and the change to a TN subtype were associated with a worse prognosis [56]. Our results documented this type of change, in which three luminal B-like HER2+ tumours lost HR expression and four luminal B-like HER2+ lost HER2 amplification. Other studies have described a post-NAT loss of HER2 amplification in 15–43% of cases [57,58]. The reason for these changes is likely related to the selective cytotoxic effect of anti-HER2 drugs on HER2+ cells, causing a selection, and consequent survival, of negative cells. Nonetheless, these results might help in finding a suitable adjuvant treatment for these non-responder patients and encourage further studies in this direction.

### 4.3. Limitations

We acknowledge that this study has some limitations. First, the relatively small number of patients included and the diversity of treatment regimens may affect the statistical analyses. Second, the limited follow-up represents a bias in our findings and longer periods of observation would strengthen our results.

## 5. Conclusions

We have demonstrated that small tumour size, lack of ER expression and high TIL values may help to predict the response of HER2+ tumours to NAT, whereas expression of PR could be a good predictor of resistance to NAT in TN tumours. Interestingly, characterisation of residual tumours showed some changes in HR and HER2 status, T-stage and Ki67 in HER2+ tumours, supporting the importance of accurate assessment of these biomarkers in residual disease. Phenotypic changes occur after NAT and this reinforces the need to reevaluate residual tumours by IHC in order to adjust post-NAT treatment. Larger studies are needed to confirm these associations, which may be of relevance in clinical practice.

## Figures and Tables

**Table 1:** Treatment regimens.

	pCR		No pCR		
	N	%	N	%	P
HER2+ (*n* = 81)					0.068
	39	48.1	42	51.9	
Anthracycline + THP	27	69.2	35	83.3	
Docetaxel + CB + TH	6	15.4	1	2.4	
THP	4	10.3	6	14.3	
THP + anti-PD-L1	2	5.1	0	0	
TN (*n* = 69)					0.794
	37	53.6	32	46.4	
P + CB + ACdd	16	43.2	15	46.9	
P + EC	18	48.6	16	50	
P + anti-PD-L1	3	8.1	1	3.1	

T = trastuzumab; H = pertuzumab; P = paclitaxel; CB = carboplatin; ACdd = adriamicine plus cyclophosphamide; E = epirubicin plus cyclophosphamide.

**Table 2:** Comparison of the histopathological characteristics between responders and non-responders.

	HER2+ (*n* = 81)					TN (*n* = 69)				
	pCR		No pCR		P	pCR		No pCR		P
	N	%	N	%		N	%	N	%	
	39	48.1	42	51.9		37	53.6	32	46.6	
Median age [range]					0.114					0.105
	57.3 [35–79]		53.6 [31–77]			49.4 [26–69]		53.7 [35–77]		
Histological subtype					>0.999					>0.999
No special	38	97.4	41	97.6		36	97.3	31	96.9	
Other	1	2.6	1	2.4		1	2.7	1	3.1	
Histological grade					0.254					0.097
Grade 1/2	18	46.2	26	61.9		4	5.8	8	25	
Grade 3	19	48.7	15	35.7		32	86.5	23	71.9	
Not available	2	5.1	1	2.4		1	2.7	1	3.1	
ER					0.022					>0.999
Negative (<1%)	19	48.7	10	23.8		35	94.6	31	96.9	
Positive (>1%)	20	51.3	32	76.2		2	5.4	1	3.1	
PR					0.471					0.743
Negative (<1%)	14	35.9	11	26.2		32	86.5	26	81.3	
Positive (>1%)	25	64.1	31	73.8		5	13.5	6	18.7	
Mean Ki67					0.022					0.224
	41.7		33.9			65		58.7		
Ki67 LI					0.195					0.201
Low 0–29%	8	20.5	13	30.9		0	0	2	6.3	
High ≥ 30%	30	76.9	22	52.4		35	94.6	27	84.4	
Not available	1	2.6	7	16.7		2	5.4	3	9.3	
Mean TILs					0.027					0.111
	23.3		14.1			27.8		18.6		
Percentage of TILs					0.032					0.617
Low ≤ 20%	25	64.1	35	83.3		22	59.5	21	65.6	
High > 20%	13	33.3	5	11.9		14	37.8	10	31.3	
Not available	1	2.6	2	4.8		1	2.7	1	3.1	
Clinical T Stage					0.005					0.496
T0/T1/T2	35	89.7	26	61.9		33	89.2	26	81.3	
T3/T4	4	10.3	16	38.1		4	10.8	6	18.7	
Clinical N Stage					0.505					0.450
Negative	18	46.2	16	38.1		25	67.6	18	56.3	
Positive	21	53.8	26	61.9		12	32.4	14	43.7	

**Table 3:** Association of variables with residual cancer burden (RCB) in HER2+ tumours.

	RCB-0/I		RCB-II/III		
	N	%	N	%	P
	55	67.9	26	32.1	
Histological grade					0.807
Grade 1/2	29	52.7	15	57.7	
Grade 3	24	43.6	10	38.5	
Not available	2	7.2	1	3.8	
ER					0.046
Negative (<1%)	24	43.6	5	19.2	
Positive (>1%)	31	56.4	21	80.8	
Not available	0	0	0	0	
PR					0.620
Negative (<1%)	18	32.7	7	26.9	
Positive (>1%)	37	67.3	19	73.1	
Not available	0	0	0	0	
Mean Ki67					0.278
	39.2		35.3		
Ki67 LI					0.395
Low (0–29%)	11	20	10	38.5	
High (≥30%)	39	70.9	13	50	
Not available	5	9.1	3	11.5	
Mean TILs					0.188
	20.4		14.7		
Percentage of TILs					0.395
Low (≤20%)	39	70.9	21	80.8	
High (>20%)	14	25.5	4	15.4	
Not available	2	3.6	1	3.8	
Clinical T Stage					0.058
T0/T1/T2	45	81.8	16	61.5	
T3/T4	10	18.2	10	38.5	
Clinical N Stage					0.473
Negative	25	45.5	9	34.6	
Positive	30	54.5	17	65.4	

**Table 4:** Association of variables with residual cancer burden (RCB) in TN tumours.

	RCB-0/I		RCB-II/III		
	N	%	N	%	P
	50	72.5	19	27.5	
Histological grade					0.281
Grade 1/2	7	14	5	26.3	
Grade 3	42	84	13	68.4	
Not available	1	2	1	5.3	
ER					0.555
Negative (<1%)	47	94	19	100	
ER-low (1–10%)	3	6	0	0	
Not available	0	0	0	0	
PR					<0.001
Negative (<1%)	44	88	8	42.1	
PR-low (1–10%)	6	12	11	57.9	
Not available	0	0	0	0	
Mean Ki67					0.706
	62.8		60.4		
Ki67 LI					0.076
Low (0–29%)	0	0	2	10.5	
High (≥30%)	46	92	16	84.2	
Not available	4	8	1	5.3	
Mean TILs					0.483
	24.7		20.3		
Percentage of TILs					0.779
Low (≤20%)	32	64	11	57.9	
High (>20%)	17	34	7	36.8	
Not available	1	2	1	5.3	
Clinical T Stage					0.445
T0/T1/T2	44	88	15	78.9	
T3/T4	6	12	4	21.1	
Clinical N Stage					0.406
Negative	33	66	10	52.6	
Positive	17	34	9	47.4	

**Table 5:** Comparison of primary and residual tumours in non-responder patients.

	HER2 (*n* = 42)						TN (*n* = 32)				
	Primary		Residual			P	Primary		Residual		P
	N	%	N	%			N	%	N	%	
Histological grade						0.206					0.086
Grade 1/2	26	61.9	25	59.5			8	25	12	37.5	
Grade 3	15	35.7	7	16.7			23	71.9	11	34.4	
Not available	1	2.4	10	23.8			1	3.1	9	28.1	
ER						0.021					>0.999
Negative (<1%)	10	23.8	11	26.2			31	96.9	28	87.5	
Positive (>1%)	32	76.2	25	59.5			1	3.1	1	3.1	
Not available	0	0	6	14.3			0	0	3	9.4	
PR						0.021					0.477
Negative (<1%)	11	26.2	19	45.5			26	81.3	26	81.3	
Positive (>1%)	31	73.8	17	40.5			6	18.7	3	9.4	
Not available	0	0	6	14.3			0	0	3	9.4	
HER2 Status						0.001					1
Negative	0	0	8		19.1		32	100	31	96.9	
Positive	42	100	28		66.6		0	0	0	0	
Not available	0	0	6		14.3		0	0	1	3.1	
Mean Ki67						<0.001					<0.001
	33.9		18.7				56		17.8		
Ki67 LI						0.007					0.201
Low (0–29%)	13	30.9	22	52.4			2	5.4	20	6.3	
High (≥30%)	22	52.4	9	21.4			27	84.4	6	84.4	
Not available	7	16.7	11	26.2			3	8.1	6	9.3	
Mean TILs						0.003					0.668
	14.1		5.2				18.6		16.5		
Percentage of TILs						0.441					1
Low (≤20%)	35		32				21		16		
High (>20%)	5		2				10		7		
Not available	2		8				1		9		
cT and ypT Stage						<0.001					<0.001
Tis/T0/T1	3	7.1	32	76.2			8	21.6	25	78.1	
T2	23	54.8	5	11.9			25	67.6	5	15.6	
T3	8	19	4	9.5			3	8.1	2	6.3	
T4	8	19	1	2.4			1	2.7	0	0	
cN and ypN Stage						0.122					0.583
Negative	16	38.1	22	52.4			18	56.3	15	46.9	
Positive	26	61.9	17	40.5			14	43.7	8	25	
Not available	0	0	3	7.1			0	0	9	28.1	

## Data Availability

The datasets generated and/or analysed during the current study are not publicly available but are available from the corresponding author on reasonable request. The policy of Hospital Universitari Germans Trias i Pujol (HUGTiP) is to share with the scientific community any data obtained in research projects, as long as ethical and legal regulations permit it. Our institution strives to publish the results, as well as supporting data in its raw, processed and analysed states, in a long-term data archive to which access may be open, or restricted, or both. HUGTiP recommends that while research is ongoing, data is stored on the institute server. For this purpose, our group has its own server space which is supported by the IT department. This server space allows for managed access to and the sharing of data between and among partners during the project. Safe and secure storage is guaranteed by the IT security and safety protocols of the institute network. If it is not possible to store the data directly on the institute network, data stored is encrypted on a local device (laptop) and transferred to the institute network as soon as possible. HUGTiP has set strict conditions for the management of research data. In accordance with the Institute’s research data management policy all research data will be archived permanently for scientific integrity reasons. All data suitable for reuse will be made available to the scientific community, together with their accompanying metadata and documentation necessary to understand the data. To this end, the services of the Research Information System of HUGTiP will be used. Via RIS, data sets are made available, a long-term data archive to which access may be open, or restricted, or both.

## References

[1] Derks M.G.M., van de Velde C.J.H. (2018). Neoadjuvant chemotherapy in breast cancer: More than just downsizing. Lancet Oncol..

[2] Mougalian S.S., Soulos P.R., Killelea B.K., Lannin D.R., Abu-Khalaf M.M., DiGiovanna M.P., Sanft T.B., Pusztai L., Gross C.P., Chagpar A.B. (2015). Use of neoadjuvant chemotherapy for patients with stage I to III breast cancer in the United States. Cancer.

[3] Clough K.B., Acosta-Marín V., Nos C., Alran S., Rouanet P., Garbay J.-R., Giard S., Verhaeghe J.-L., Houvenaeghel G., Flipo B. (2015). Rates of Neoadjuvant Chemotherapy and Oncoplastic Surgery for Breast Cancer Surgery: A French National Survey. Ann. Surg. Oncol..

[4] Vugts G., Maaskant-Braat A.J.G., Nieuwenhuijzen G.A.P., Roumen R.M.H., Luiten E.J.T., Voogd A.C. (2016). Patterns of Care in the Administration of Neo-adjuvant Chemotherapy for Breast Cancer. A Population-Based Study. Breast J..

[5] Von Minckwitz G., Untch M., Blohmer J.-U., Costa S.D., Eidtmann H., Fasching P.A., Gerber B., Eiermann W., Hilfrich J., Huober J. (2012). Definition and Impact of Pathologic Complete Response on Prognosis After Neoadjuvant Chemotherapy in Various Intrinsic Breast Cancer Subtypes. J. Clin. Oncol..

[6] Cortazar P., Geyer C.E. (2015). Pathological Complete Response in Neoadjuvant Treatment of Breast Cancer. Ann. Surg. Oncol..

[7] Spring L., Greenup R., Niemierko A., Schapira L., Haddad S., Jimenez R., Coopey S., Taghian A., Hughes K.S., Isakoff S.J. (2017). Pathologic Complete Response After Neoadjuvant Chemotherapy and Long-Term Outcomes Among Young Women with Breast Cancer. J. Natl. Compr. Cancer Netw..

[8] Untch M., Möbus V., Kuhn W., Muck B.R., Thomssen C., Bauerfeind I., Harbeck N., Werner C., Lebeau A., Schneeweiss A. (2009). Intensive Dose-Dense Compared with Conventionally Scheduled Preoperative Chemotherapy for High-Risk Primary Breast Cancer. J. Clin. Oncol..

[9] Schneeweiss A., Chia S., Hickish T., Harvey V., Eniu A., Hegg R., Tausch C., Seo J.H., Tsai Y.-F., Ratnayake J. (2013). Pertuzumab plus trastuzumab in combination with standard neoadjuvant anthracycline-containing and anthracycline-free chemotherapy regimens in patients with HER2-positive early breast cancer: A randomized phase II cardiac safety study (TRYPHAENA). Ann. Oncol..

[10] Swain S.M., Ewer M.S., Viale G., Delaloge S., Ferrero J.-M., Verrill M., Colomer R., Vieira C., Werner T.L., Douthwaite H. (2018). Pertuzumab, trastuzumab, and standard anthracycline- and taxane-based chemotherapy for the neoadjuvant treatment of patients with HER2-positive localized breast cancer (BERENICE): A phase II, open-label, multicenter, multinational cardiac safety study. Ann. Oncol..

[11] Denkert C., Von Minckwitz G., Darb-Esfahani S., Lederer B., Heppner B.I., Weber K.E., Budczies J., Huober J., Klauschen F., Furlanetto J. (2018). Tumour-infiltrating lymphocytes and prognosis in different subtypes of breast cancer: A pooled analysis of 3771 patients treated with neoadjuvant therapy. Lancet Oncol..

[12] Kurozumi S., Inoue K., Takei H., Matsumoto H., Kurosumi M., Horiguchi J., Takeyoshi I., Oyama T. (2015). ER, PgR, Ki67, p27Kip1, and histological grade as predictors of pathological complete response in patients with HER2-positive breast cancer receiving neoadjuvant chemotherapy using taxanes followed by fluorouracil, epirubicin, and cyclophosphamide concomitant with trastuzumab. BMC Cancer.

[13] Alba E., Lluch A., Ribelles N., Anton-Torres A., Sanchez-Rovira P., Albanell J., Calvo L., García-Asenjo J.A.L., Palacios J., Chacon J.I. (2016). High Proliferation Predicts Pathological Complete Response to Neoadjuvant Chemotherapy in Early Breast Cancer. Oncologist.

[14] Goorts B., Van Nijnatten T.J.A., De Munck L., Moossdorff M., Heuts E.M., De Boer M., Lobbes M.B.I., Smidt M.L. (2017). Clinical tumor stage is the most important predictor of pathological complete response rate after neoadjuvant chemotherapy in breast cancer patients. Breast Cancer Res. Treat..

[15] Amin M.B., Greene F.L., Edge S.B., Compton C.C., Gershenwald J.E., Brookland R.K., Meyer L., Gress D.M., Byrd D.R., Winchester D.P. (2017). The Eighth Edition AJCC Cancer Staging Manual: Continuing to build a bridge from a population-based to a more “personalized” approach to cancer staging. CA Cancer J. Clin..

[16] Symmans W.F., Wei C., Gould R., Yu X., Zhang Y., Liu M., Walls A., Bousamra A., Ramineni M., Sinn B. (2017). Long-Term Prognostic Risk After Neoadjuvant Chemotherapy Associated with Residual Cancer Burden and Breast Cancer Subtype. J. Clin. Oncol..

[17] Symmans W.F., Peintinger F., Hatzis C., Rajan R., Kuerer H., Valero V., Assad L., Poniecka A., Hennessy B., Green M. (2007). Measurement of Residual Breast Cancer Burden to Predict Survival After Neoadjuvant Chemotherapy. J. Clin. Oncol..

[18] Wolff A.C., Hammond M.E.H., Allison K.H., Harvey B.E., Mangu P.B., Bartlett J.M.S., Bilous M., Ellis I.O., Fitzgibbons P., Hanna W. (2018). Human Epidermal Growth Factor Receptor 2 Testing in Breast Cancer: American Society of Clinical Oncology/College of American Pathologists Clinical Practice Guideline Focused Update. J. Clin. Oncol..

[19] Nielsen T.O., Leung S.C.Y., Rimm D.L., Dodson A., Acs B., Badve S., Denkert C., Ellis M.J., Fineberg S., Flowers M. (2021). Assessment of Ki67 in Breast Cancer: Updated Recommendations from the International Ki67 in Breast Cancer Working Group. Gynecol. Oncol..

[20] Curigliano G., Burstein H.J., Winer E.P., Gnant M., Dubsky P., Loibl S., Colleoni M., Regan M.M., Piccart-Gebhart M., Senn H.-J. (2017). De-escalating and escalating treatments for early-stage breast cancer: The St. Gallen International Expert Consensus Conference on the Primary Therapy of Early Breast Cancer 2017. Ann. Oncol. Off. J. Eur. Soc. Med. Oncol..

[21] Zhu X., Chen L., Huang B., Wang Y., Ji L., Wu J., Di G., Liu G., Yu K., Shao Z. (2020). The prognostic and predictive potential of Ki-67 in triple-negative breast cancer. Sci. Rep..

[22] Salgado R., Denkert C., Demaria S., Sirtaine N., Klauschen F., Pruneri G., Wienert S., Van den Eynden G., Baehner F.L., Penault-Llorca F. (2015). The evaluation of tumor-infiltrating lymphocytes (TILs) in breast cancer: Recommendations by an International TILs Working Group 2014. Ann. Oncol..

[23] Dieci M.V., Radosevic-Robin N., Fineberg S., Eynden G.v.D., Ternes N., Penault-Llorca F., Pruneri G., D’alfonso T.M., Demaria S., Castaneda C. (2017). Update on tumor-infiltrating lymphocytes (TILs) in breast cancer, including recommendations to assess TILs in residual disease after neoadjuvant therapy and in carcinoma in situ: A report of the International Immuno-Oncology Biomarker Working Group on Breast Cancer. Semin. Cancer Biol..

[24] (2005). Early Breast Cancer Trialists’ Collaborative Group (EBCTCG) Effects of chemotherapy and hormonal therapy for early breast cancer on recurrence and 15-year survival: An overview of the randomised trials. Lancet.

[25] Jarząb M., Stobiecka E., Badora-Rybicka A., Chmielik E., Kowalska M., Bal W., Polakiewicz-Gilowska A., Bobek-Billewicz B., Lange D., Tarnawski R. (2019). Association of breast cancer grade with response to neoadjuvant chemotherapy assessed postoperatively. Pol. J. Pathol..

[26] Díaz-Redondo T., Lavado-Valenzuela R., Jimenez B., Pascual T., Gálvez F., Falcón A., Alamo M.D.C., Morales C., Amerigo M., Pascual J. (2019). Different Pathological Complete Response Rates According to PAM50 Subtype in HER2+ Breast Cancer Patients Treated With Neoadjuvant Pertuzumab/Trastuzumab vs. Trastuzumab Plus Standard Chemotherapy: An Analysis of Real-World Data. Front. Oncol..

[27] Gass P., Lux M.P., Rauh C., Hein A., Bani M.R., Fiessler C., Hartmann A., Häberle L., Pretscher J., Erber R. (2018). Prediction of pathological complete response and prognosis in patients with neoadjuvant treatment for triple-negative breast cancer. BMC Cancer.

[28] Meisel J.L., Zhao J., Suo A., Zhang C., Wei Z., Taylor C., Aneja R., Krishnamurti U., Li Z., Nahta R. (2020). Clinicopathologic Factors Associated with Response to Neoadjuvant Anti-HER2–Directed Chemotherapy in HER2-Positive Breast Cancer. Clin. Breast Cancer.

[29] Ács B., Zámbó V., Vízkeleti L., Szász A.M., Madaras L., Szentmártoni G., Tőkés T., Molnár B., Molnár I.A., Vári-Kakas S. (2017). Ki-67 as a controversial predictive and prognostic marker in breast cancer patients treated with neoadjuvant chemotherapy. Diagn. Pathol..

[30] Chen X., He C., Han D., Zhou M., Wang Q., Tian J., Li L., Xu F., Zhou E., Yang K. (2017). The predictive value of Ki-67 before neoadjuvant chemotherapy for breast cancer: A systematic review and meta-analysis. Futur. Oncol..

[31] Zhang G., Xie W., Liu Z., Lin C., Piao Y., Xu L., Guo F., Xie X. (2014). Prognostic function of Ki-67 for pathological complete response rate of neoadjuvant chemotherapy in triple-negative breast cancer. Tumori J..

[32] Sánchez-Muñoz A., Plata-Fernández Y.M., Fernández M., Jaén-Morago A., Fernández-Navarro M., de la Torre-Cabrera C., Ramirez-Tortosa C., Lomas-Garrido M., Llácer C., Navarro-Perez V. (2013). The Role of Immunohistochemistry in Breast Cancer Patients Treated With Neoadjuvant Chemotherapy: An Old Tool With an Enduring Prognostic Value. Clin. Breast Cancer.

[33] Wajid S., Samad F.A., Syed A.S., Kazi F. (2021). Ki-67 and Its Relation with Complete Pathological Response in Patients With Breast Cancer. Cureus.

[34] Tao M., Chen S., Zhang X., Zhou Q. (2017). Ki-67 labeling index is a predictive marker for a pathological complete response to neoadjuvant chemotherapy in breast cancer. Medicine.

[35] Tan S., Fu X., Xu S., Qiu P., Lv Z., Xu Y., Zhang Q. (2021). Quantification of Ki67 Change as a Valid Prognostic Indicator of Luminal B Type Breast Cancer After Neoadjuvant Therapy. Pathol. Oncol. Res..

[36] Pistelli M., Merloni F., Crocetti S., Scortichini L., Tassone L., Cantini L., Agostinelli V., Bastianelli L., Savini A., Berardi R. (2021). Prognostic Impact of Ki-67 Change in Locally Advanced and Early Breast Cancer after Neoadjuvant Chemotherapy: A Single Institution Experience. J. Oncol..

[37] Li L., Han N., Wang X., Wang Q., Tian J., Yao J., Yuan L., Qian K., Zou Q., Yi W. (2017). Prognostic values of Ki-67 in neoadjuvant setting for breast cancer: A systematic review and meta-analysis. Futur. Oncol..

[38] Miglietta L., Morabito F., Provinciali N., Canobbio L., Meszaros P., Naso C., Murialdo R., Boitano M., Salvi S., Ferrarini M. (2013). A prognostic model based on combining estrogen receptor expression and Ki-67 value after neoadjuvant chemotherapy predicts clinical outcome in locally advanced breast cancer: Extension and analysis of a previously reported cohort of patients. Eur. J. Surg. Oncol. (EJSO).

[39] Liu Y., Yin W., Yan T., Du Y., Shao Z., Lu J. (2013). The clinical significance of Ki-67 as a marker of prognostic value and chemosensitivity prediction in hormone-receptor-positive breast cancer: A meta-analysis of the published literature. Curr. Med. Res. Opin..

[40] Denkert C., Loibl S., Noske A., Roller M., Müller B.M., Komor M., Budczies J., Darb-Esfahani S., Kronenwett R., Hanusch C. (2010). Tumor-Associated Lymphocytes as an Independent Predictor of Response to Neoadjuvant Chemotherapy in Breast Cancer. J. Clin. Oncol..

[41] Lee H., Lee M., Seo J.-H., Gong G., Lee H.J. (2020). Changes in Tumor-infiltrating Lymphocytes After Neoadjuvant Chemotherapy and Clinical Significance in Triple Negative Breast Cancer. Anticancer Res..

[42] Caudle A.S., Gonzalez-Angulo A.M., Hunt K.K., Liu P., Pusztai L., Symmans W.F., Kuerer H.M., Mittendorf E.A., Hortobagyi G.N., Meric-Bernstam F. (2010). Predictors of Tumor Progression During Neoadjuvant Chemotherapy in Breast Cancer. J. Clin. Oncol..

[43] Jin X., Jiang Y.-Z., Chen S., Yu K.-D., Ma D., Sun W., Shao Z.-M., Di G.-H. (2016). A nomogram for predicting pathological complete response in patients with human epidermal growth factor receptor 2 negative breast cancer. BMC Cancer.

[44] Prat A., Fan C., Fernández A., Hoadley K.A., Martinello R., Vidal M., Viladot M., Pineda E., Arance A., Muñoz M. (2015). Response and survival of breast cancer intrinsic subtypes following multi-agent neoadjuvant chemotherapy. BMC Med..

[45] Taucher S., Rudas M., Gnant M., Thomanek K., Dubsky P., Roka S., Bachleitner-Hofmann T., Kandioler D., Wenzel C., Steger G. (2003). Sequential steroid hormone receptor measurements in primary breast cancer with and without intervening primary chemotherapy. Endocr. Relat. Cancer.

[46] Kasami M., Uematsu T., Honda M., Yabuzaki T., Sanuki J., Uchida Y., Sugimura H. (2008). Comparison of estrogen receptor, progesterone receptor and Her-2 status in breast cancer pre- and post-neoadjuvant chemotherapy. Breast.

[47] Mittendorf E.A., Wu Y., Scaltriti M., Meric-Bernstam F., Hunt K.K., Dawood S., Esteva F.J., Buzdar A.U., Chen H., Eksambi S. (2009). Loss of *HER2* Amplification Following Trastuzumab-Based Neoadjuvant Systemic Therapy and Survival Outcomes. Clin. Cancer Res..

[48] Hurley J., Doliny P., Reis I., Silva O., Gomez-Fernandez C., Velez P., Pauletti G., Powell J.E., Pegram M.D., Slamon D.J. (2006). Docetaxel, Cisplatin, and Trastuzumab as Primary Systemic Therapy for Human Epidermal Growth Factor Receptor 2–Positive Locally Advanced Breast Cancer. J. Clin. Oncol..

[49] Harris L.N., You F., Schnitt S.J., Witkiewicz A., Lu X., Sgroi D., Ryan P.D., Come S.E., Burstein H.J., Lesnikoski B.-A. (2007). Predictors of Resistance to Preoperative Trastuzumab and Vinorelbine for HER2-Positive Early Breast Cancer. Clin. Cancer Res..

[50] Grassini D., Cascardi E., Sarotto I., Annaratone L., Sapino A., Berrino E., Marchiò C. (2022). Unusual Patterns of HER2 Expression in Breast Cancer: Insights and Perspectives. Pathobiology.

[51] Matsubara N., Mukai H., Fujii S., Wada N. (2012). Different prognostic significance of Ki-67 change between pre- and post-neoadjuvant chemotherapy in various subtypes of breast cancer. Breast Cancer Res. Treat..

[52] Chen C., Zhang Y., Huang Z., Wu J., Huang W., Zhang G. (2019). Decrease in the Ki67 index during neoadjuvant chemotherapy predicts favorable relapse-free survival in patients with locally advanced breast cancer. Cancer Biol. Med..

[53] Ding Y., Ding K., Qian H., Yu X., Zou D., Yang H., Mo W., He X., Zhang F., Qin C. (2020). Impact on survival of estrogen receptor, progesterone receptor and Ki-67 expression discordance pre- and post-neoadjuvant chemotherapy in breast cancer. PLoS ONE.

[54] Wang Y., Zong B., Yu Y., Wang Y., Tang Z., Chen R., Huang M., Liu S. (2021). Ki67 Index Changes and Tumor-Infiltrating Lymphocyte Levels Impact the Prognosis of Triple-Negative Breast Cancer Patients with Residual Disease After Neoadjuvant Chemotherapy. Front. Oncol..

[55] Ochi T., Bianchini G., Ando M., Nozaki F., Kobayashi D., Criscitiello C., Curigliano G., Iwamoto T., Niikura N., Takei H. (2019). Predictive and prognostic value of stromal tumour-infiltrating lymphocytes before and after neoadjuvant therapy in triple negative and HER2-positive breast cancer. Eur. J. Cancer.

[56] Jin X., Jiang Y.-Z., Chen S., Yu K.-D., Shao Z.-M., Di G.-H. (2015). Prognostic value of receptor conversion after neoadjuvant chemotherapy in breast cancer patients: A prospective observational study. Oncotarget.

[57] Yang Y.-F., Liao Y.-Y., Li L.-Q., Xie S.-R., Xie Y.-F., Peng N.-F. (2013). Changes in ER, PR and HER2 receptors status after neoadjuvant chemotherapy in breast cancer. Pathol. Res. Pract..

[58] Van De Ven S., Smit V.T.H.B.M., Dekker T.J.A., Nortier J.W.R., Kroep J.R. (2011). Discordances in ER, PR and HER2 receptors after neoadjuvant chemotherapy in breast cancer. Cancer Treat. Rev..

